# Integrated Transcriptomic and Epigenetic Study of PCOS: Impact of *Map3k1* and *Map1lc3a* Promoter Methylation on Autophagy

**DOI:** 10.3389/fgene.2021.620241

**Published:** 2021-03-08

**Authors:** Yulan Qin, Ting Li, Hui Zhao, Zhanrui Mao, Chunxia Ding, Yani Kang

**Affiliations:** ^1^School of Biomedical Engineering, Bio-ID Center, Shanghai Jiao Tong University, Shanghai, China; ^2^Department of Obstetrics and Gynecology, Yuncheng Central Hospital, Yuncheng, China

**Keywords:** polycystic ovary syndrome, PNA mice, DNA methylation, MAPK pathway, autophagy

## Abstract

Polycystic ovary syndrome (PCOS) is a prevalent heterogeneous endocrine and metabolic disorder in women of reproductive age. Epigenetic mechanisms contribute to the development of PCOS. Nevertheless, the role of DNA methylation in the development of PCOS remains unclear. To investigate the molecular mechanisms underlying the hyperandrogenic phenotype of PCOS, dihydrotestosterone (DHT)-induced prenatally androgenized (PNA) mice were used to mimic this phenotype. Ovarian samples from PNA and control mice were subjected to methyl-CpG-binding domain (MBD)-seq and RNA-seq, and validation was conducted using methylation-specific polymerase chain reaction (MSP) and quantitative real-time PCR (RT-qPCR). Immunohistochemical analysis (using anti-LC3II antibody) and transmission electron microscopy were conducted using ovarian tissue sections (which included granulosa cells) from PNA and control mice. There were 857 genes with differentially methylated promoter regions and 3,317 differentially expressed genes (DEGs) in the PNA mice compared to the control mice. Downregulation of *Dnmt1* (which encodes DNA methyltransferase 1), accompanied by global hypomethylation, was observed in the PNA mice compared to the control mice. The promoter regions of *Map3k1* (which encodes MEKK1) and *Map1lc3a* (which encodes LC3II) were hypomethylated, accompanied by upregulation of *Map3k1* and *Map1lc3a* mRNA expression. The autophagy profiling results showed that LC3II protein expression and autophagosomes were significantly increased in the granulosa cells of PNA mice. Additionally, the mRNA expression of genes related to the mitogen-activated protein kinase (MAPK)/p53 pathway (*Mapk14*, *Mapkapk3*, and *Trp53*) and the autophagy-related gene *Becn1* were significantly increased. DHT could change the DNA methylation and transcription level of *Map3k1* and lead to an activation of autophagy in granulosa cells. These observations indicated that the change in autophagy may be driven by MAPK/p53 pathway activation, which may have been caused by DHT-induced transcriptional, and the methylation level changed of the key upstream gene *Map3k1*. Our study provides a novel genetic basis and new insights regarding the pathogenesis of PCOS.

## Introduction

Polycystic ovary syndrome (PCOS) is one of the most common endocrine diseases, with features such as chronic anovulation, hyperandrogenism, and polycystic ovaries (Franks, 1995). It is associated with an increased risk of metabolic aberrations and other conditions, including hyperinsulinism, insulin resistance, dyslipidemia, type 2 diabetes mellitus, and endometrial carcinoma. Along with being related to symptoms such as acne and obesity, it is also a common cause of ovarian dysfunction in women, involving menstrual dysfunction and infertility. In addition to genetic predisposition, environmental factors, and lifestyle factors, emerging evidence suggests that epigenetic modification/regulation contributes to the development of PCOS (Azziz et al., 2004; Qu et al., 2012). DNA methylation is an important epigenetic modification affecting the regulation of gene expression. Aberrant DNA methylation manifests in both preservation of global genome stability and localized gene promoter changes, which influence the transcription of disease-causing genes (Das et al., 2008). Recent research has demonstrated significant changes in DNA methylation and transcription patterns in the ovaries (Yu et al., 2015) including the granulosa cells (Xu et al., 2016) and adipose tissue of patients with PCOS (Kokosar et al., 2016). The abnormal DNA methylation of estrogen and androgen synthesis-related genes (such as *NCOR1*, *PPARG1*, *HDAC3*, and *CYP19A1*) is believed to be a major factor underlying the development of hyperandrogenemia in PCOS (Hosseini et al., 2019). Hyperandrogenism is one of the main features, and it is included in the clinical diagnostic criteria for PCOS. Rather than directly assessing the complex regulatory network of the human body, animal models can help to investigate the pathophysiologic mechanisms underlying certain phenotypes of PCOS. To mimic the hyperandrogenic phenotype of PCOS patients, we subcutaneously injected dihydrotestosterone (DHT) into pregnant mice to create a uterine environment involving high androgen levels and to induce hyperandrogenemia in the offspring. Prenatally androgenized (PNA) mice are commonly used in serological, pathological, and omics-related studies of the hyperandrogenic phenotype of PCOS.

We identified differentially methylated genes (DMGs) that had differentially methylated promoter regions by carrying out global methyl-CpG-binding domain (MBD)-seq, and we identified differentially expressed genes (DEGs) by carrying out RNA-seq, in the ovarian tissues of PNA and control mice. We then identified the significant biological pathways related to the DMGs and DEGs. Selected genes were validated using methylation-specific polymerase chain reaction (MSP) and quantitative real-time PCR (RT-qPCR). Furthermore, autophagy profiling, involving immunohistochemical analysis and transmission electron microscopy (TEM), was performed in the PNA and control mice. Furthermore, we treated granular cells with DHT *in vitro* to verify the direct effect of androgens on the cells. Our aim was to investigate the potential involvement of epigenetic dysregulation in the pathogenesis of PCOS in mice.

## Materials and Methods

### Construction of PNA Mouse Model of PCOS

All of the experimental procedures were performed in accordance with the guidelines of the Experimental Animals Management Committee (Jiangsu Province, China) and were approved by the Nanjing Drum Tower Hospital Experimental Animals Welfare and Ethical Committee (no. 20150302). Adult Institute of Cancer Research (ICR) mice were purchased from the Animal Experimental Center of Yangzhou University (Jiangsu Province, China). They were housed at the Drum Tower Hospital Animal Experimental Center (Jiangsu Province, China) at a constant temperature, humidity, and light duration and with *ad libitum* access to chow and water.

Females were mated with males and checked for copulatory plugs daily. The date of the plug development was considered to represent day 1 of gestation. On days 16–18 of gestation, pregnant dams were subcutaneously injected daily with 70 μl sesame oil containing 350 μg DHT or sesame oil vehicle. The offspring born in the DHT group were considered to be PNA mice, and the offspring born in the control group were considered to be control mice. All offspring were housed in specific-pathogen-free animal rooms. The mice were euthanized using the anesthetic chloral hydrate, and ovarian tissues were subsequently harvested. The DHT-induced PNA mouse model from that laboratory was used in some research, and the data on metabolic, hormonal, and reproductive/ovarian parameters for the PNA mouse model have been previously published (Lei et al., 2017).

### Confirmation of Model Establishment and Selection of Mice

Mice that were significantly different regarding body weight were excluded. To confirm the establishment of the PCOS model, the number of secondary follicles, serum testosterone level, and estrus duration were assessed at 8 weeks of age. From among the female mice, we then randomly selected six control mice and six PNA mice to be assessed in this study. The data of model assessment including the number of secondary follicles, serum testosterone level, and estrus duration are shown in Table 1 and Supplementary Table S1.

**TABLE 1:** Identification data of prenatal androgenized (PNA) mice and control subjects.

**PNA mice characteristics**	**PNA mice (*n* = 6)**	**Normal (*n* = 6)**	***p*-value**
Age (weeks)	8 weeks	8 weeks	/
Proportion of estrus duration	0.35 ± 0.05	0.18 ± 0.07	0.0014
Number of secondary follicles	687 ± 145	402 ± 74	0.0016
Testosterone (nmol/L)	226.89 ± 13.90	187.45 ± 9.44	0.0002

*All data are presented as the mean ± SEM. *p* < 0.05 was considered significant.*

### Ovarian Tissue Processing and Histological Examination of Ovary Morphology

The ovaries were dissected, weighed, fixed in 4% paraformaldehyde at 4°C overnight, and then stored in 70% alcohol until histological processing. They were then dehydrated using alcohol, cleared using xylene, embedded in paraffin, sectioned at 5 μm thickness, mounted onto 3-aminopropyltriethoxysilane-coated glass slides, and then dried overnight in a 50°C incubator or incubated at 60°C for 2 h (Burchall et al., 2019). The serial sections were then stained with hematoxylin and eosin (HE) to observe follicular development and to count the number of follicles. The follicles of various maturation stages (including secondary follicles) were counted under high magnification. The assessor was blinded to the treatment group, and they counted the number of follicles in every fifth section, and the number obtained was multiplied by five.

### Estrus Cycle and Serological Identification

To assess estrous duration, at 8 weeks of age, the mice underwent daily vaginal smears for 2 weeks or at least one complete estrous cycle. To assess the serum testosterone level, each mouse in diestrus was anesthetized with chloral hydrate, an eyeball was removed, and blood was collected. The blood was left at room temperature for 20 min and then centrifuged at 1,000 *g* for 10 min. The supernatant was then collected and frozen at −80°C. After thawing on ice, the serum testosterone level was assessed using an enzyme-linked immunosorbent assay (ELISA) kit (Beckman Coulter, Inc., United States) and a microplate reader (BioTek, Inc., Winooski, VT, United States).

### Total RNA and Genomic DNA Extraction

Mouse ovarian tissues were removed from a refrigerator at −80°C for grounding with liquid nitrogen. Total RNA was extracted from ovarian tissue samples by using TRIzol reagents (Invitrogen, Waltham, MA, United States) as recommended by the manufacturer. The total RNA quantity was determined with Nanodrop 2000 (Thermo Scientific, Waltham, MA, United States), and quality was assessed by running 1.5% agarose gel electrophoresis and staining with 4S Red Plus Nucleic Acid Stain (Sango, Shanghai, China).

Genomic DNA from ovarian tissues of PNA mice and control group mice was extracted by using the QIAamp DNA Mini Kit (Qiagen, Hilden, Germany) following the manufacturer’s protocol.

### MBD Sequencing and Bioinformatics Analysis

To identify the DMRs in the PNA mice, ovarian tissues from three PNA mice and two control mice were assessed by MBD-seq, followed by a comparative analysis of the methylated regions. Up to 1 μg genomic DNA was fragmented with M220 Focused-ultrasonicator (Covaris, Woburn, MA, United States). The fragmented DNA with methylated CpGs was enriched by using the MethylMiner^TM^ Kit (Invitrogen, Waltham, MA, United States) as recommended by the manufacturer. The MBD-enriched DNA fractions were used to generate indexed libraries with the NEBNext Ultra DNA Library Prep Kit for Illumina (NEB, San Diego, CA, United States). The quality of libraries was assessed with Agilent 2100 Bioanalyzer (Agilent, United States).

MBD-seq reads with low-quality data filtered out were mapped to the reference genome using bowtie2 (Langmead and Salzberg, 2012). Then, SAM files were converted to sorted BAM files under the constraint of MAPQ30. The MEDIPS package (version 1.24.0) was used for the analysis and comparison of DNA methylation datasets of PCOS and controls (Lienhard et al., 2014). *P* values < 0.05 and | log_2_FC| ≥ 1 were considered to show differentially methylated regions. Raw sequence data of MBD-seq were submitted to the GEO database^1^ and represented under one super-series along with transcriptome sequencing (Accession Number: GSE156961).

### Transcriptome Sequencing and Data Analysis

To determine the significant DEGs between the PNA and control mice, the transcriptome profiles of ovaries from three PNA mice and two control mice were assessed by RNA-seq; 1 μg of total RNA was used to construct the RNA-seq libraries by the KAPA Stranded RNA-Seq Library Preparation Kit (KAPA Biosystems, Wilmington, MA, United States). The quality of libraries was assessed with Agilent 2100 Bioanalyzer (Agilent, United States).

Sequencing reads were aligned to the UCSC Mouse Genome Browser assembly with HISAT2 (version 2.0.5), after data preprocessing (Kim et al., 2019). Stringtie (version 1.3.3) was then used to determine the read counts per gene based on Ensembl gene-level annotations (Mouse GRCm38/mm10) (Pertea et al., 2016). The final unnormalized counts were assembled into a count matrix with R (version 3.6.0), and this served as an input for DESeq2 (version 1.24.0) and edgeR (version 3.26.5) (Robinson et al., 2010; Love et al., 2014). In this study, the intersections of DEGs identified by DESeq2 and edgeR were used for further analysis, and expression changes with of | log_2_FC| ≥ 1 and *p*-values < 0.05 were considered significant.

### GCs Isolation and DHT Treatment

Ovaries from six-week-old female ICR mice were placed in DMEM/F12 medium (Gibco BRL/Invitrogen, Carlsbad, CA, United States) containing 10% fetal bovine serum (HyClone, South Logan, UT, United States), 1 mM sodium pyruvate, 2 mM glutamine, 100 IU/ml penicillin, and 100 μg/ml streptomycin; 1-ml syringe needles were used to puncture the ovaries and release the oocytes and granulosa cells. Then, the oocytes were filtered out by a 40-μm cell strainer to collect the pure granulosa cells. In the experiment group, GCs were cultured in a medium supplemented with DHT (20649, MCE, China). The control group was cultured in the medium without DHT. All samples were incubated at 37°C and 5% CO2. Total RNA, genomic DNA, and total protein were extracted from these granulosa cells.

### Validation of the Methylation Level and DEGs Expression by MSP and RT-qPCR

To investigate the regulatory relationship between the MAPK pathway and autophagy in PNA mice’s ovaries, we performed MSP and RT-qPCR in the ovarian tissues of three PNA mice and three control mice. Moreover, the same detections were performed on DHT-treated ovarian granulosa cells and the control group *in vitro*. EZ DNA Methylation-Gold^TM^ Kit was used to conduct bisulfite conversion on the genomic DNA of PNA mouse ovarian tissues and the control group. Then, PCR was performed by using primers designed for hypermethylated and hypomethylated DNA amplification, respectively. Primer sequences are shown in Supplementary Table S2A.

Five hundred nanograms of total RNA was used for reverse transcription by using the PrimeScript RT Master Mix (TAKARA), which contained a mixture of random 6-mers and an oligo dT primer. RT-qPCR was carried out with a PowerUp SYBR Green Master Mix (Applied Biosystems) using the StepOne Plus qPCR instrument (Applied Biosystems). Primer sequences are shown in Supplementary Table S2A.

### Validation of the LC3II Expression by Immunohistochemistry

To investigate changes in autophagy in PNA mice’s ovaries, we performed immunohistochemical staining using the anti-LC3II antibody in the ovarian tissues of three PNA mice and three control mice. Briefly, serial 4-μm sections were obtained from each paraffin-embedded tissue block. Following deparaffinization and rehydration, sections were subjected to microwave antigen retrieval. The sections were incubated in goat serum for 1 h at room temperature, followed by anti-LC3II (1:300, ab48394, Abcam, United Kingdom), and the sections were incubated overnight at 4°C with this antibody. 3,3′-Diaminobenzidine was used as the chromogen. Nuclei were counterstained with hematoxylin, and slides were dried and mounted.

### Transmission Electron Microscopy of Autophagosomes

Furthermore, we performed TEM in the granulosa cells of three PNA mice and three control mice to investigate autophagosome change in PNA mice’s ovaries. Ovaries from PNA mice and the control group were placed in a DMEM/F12 medium (Gibco BRL/Invitrogen, Carlsbad, CA, United States) containing 10% fetal bovine serum (HyClone, South Logan, UT, United States), 2 mM glutamine, 1 mM sodium pyruvate, 100 IU/ml penicillin, and 100 μg/ml streptomycin; 1-ml syringe needles were used to puncture the ovaries for releasing the granulosa cells, then the oocytes were filtered by a 40-μm cell strainer. The isolated granulosa cells were fixed with 2.5% glutaraldehyde for more than 6 h at 4°C and fixed in 1% osmium tetroxide in the above buffer for 1 h at room temperature. The samples were embedded in Epon after being dehydrated through a graded series of ethanol; 2% uranyl acetate and lead citrate were used for ultrathin sections staining. The TEM was performed using a FEI Tecnai G2 Spirit Biotwin transmission Electron Microscope (FEI Tecnai G2, America).

### Western Blot

Total protein was extracted from mGCs cells, which were then separated *via* SDS/PAGE. The samples were transferred to a polyvinylidene difluoride (PVDF) membrane. Afterward, the membrane was incubated with primary antibodies anti-LC3B (1:1000, ab48394, Abcam, United Kingdom), followed by a peroxidase-conjugated secondary antibody. Then, an ECL detection kit (Amersham Pharmacia Biosciences) was applied for visualization. The data were quantified and normalized to Actin (1:1,000, ab8226, Abcam, United Kingdom).

### Statistical Analysis

Statistical analyses were conducted by using Statistical Package for Social Sciences (SPSS) version 20.0 software (IBM Corp., Armonk, NY, United States). The differences between groups were assessed by two-sided Student’s *t*-test, and a *p*-value < 0.05 was considered statistically significant.

## Results

### Ovary Morphology and Number of Secondary Follicles

**FIGURE 1:**
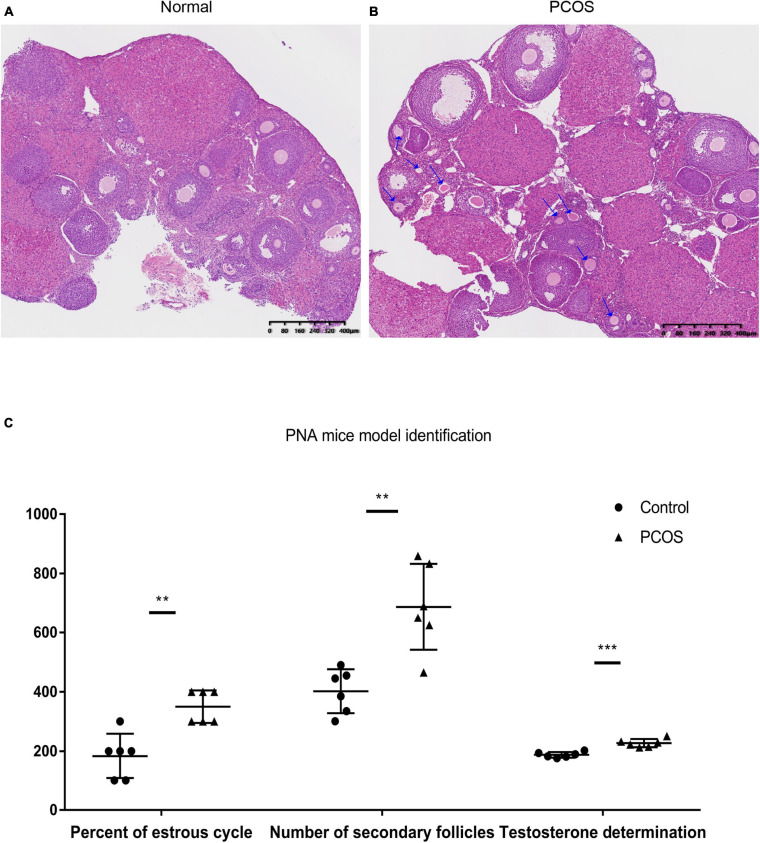
Histological and serological identification of normal (control) and PNA mice ovaries. **(A)** HE-stained ovarian section of the control group. **(B)** HE-stained ovarian section of PNA mice; the arrows mark the original follicles. **(C)** Determination of serum testosterone, number of secondary follicles, and proportion of estrus in PNA mice and control group (**p < 0.01, and ***p < 0.001; the proportion of estrus data multiplied by 100 to ensure the height of the ordinate with no change of *p*-value).

The ovaries of the DHT-induced PNA mice exhibited the classic polycystic ovary morphology, with fewer corpora lutea, indicating rare ovulation. In contrast, the control ovaries displayed numerous corpora lutea, consistent with recent ovulation. Moreover, follicles with an atretic cyst-like appearance were observed in ovaries from the PNA mice, but not from the control mice (Figures 1A,B). Additionally, the number of secondary follicles was significantly higher in the PNA mice (687 ± 145; *n* = 6) than in the control mice (402 ± 74; *n* = 6; *p* < 0.01).

### Estrous Duration and Serum Testosterone Levels

To examine the endocrine factors contributing to abnormal reproductive cycles in PNA mice, the serum testosterone level was measured, which was significantly higher in the PNA mice (226.89 ± 13.90; *n* = 6) than in the control mice (187.45 ± 9.44; *n* = 6; *p* < 0.001). Moreover, the estrous duration was significantly longer in the PNA mice (0.35 ± 0.05; *n* = 6) than in the control mice (0.18 ± 0.07; *n* = 6; *p* < 0.01). The results are consistent with the characteristics of PNA mice model in previous research (Figure 1C).

### Identification of Differentially Methylated Regions and Functional Enrichment Analyses

**FIGURE 2:**
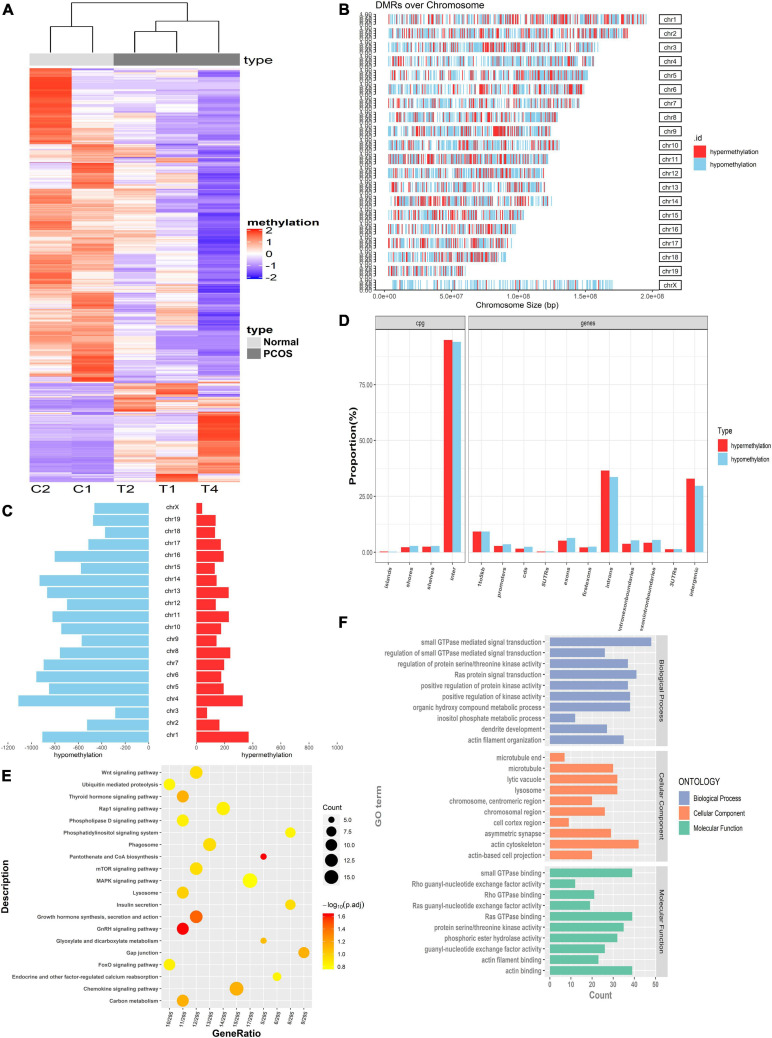
Identification of DMRs and function analysis between PNA mice and controls. **(A)** The hierarchical clustering heat map of DMR in PNA mice (T1, T2, and T4) vs. control group (C1 and C2). **(B)** Representation of the distribution of hypermethylated (red) and hypomethylated (blue) regions across whole genomes. **(C)** Histogram of hypermethylated (red) and hypomethylated (blue) region distribution over chromosomes. **(D)** The distribution of hypermethylated and hypomethylated DMRs located in genome feature in PNA mice vs. control groups. **(E)** KEGG analysis of genes with the hypermethylated promoters. **(F)** GO analysis of genes with the hypermethylated promoters.

To identify the DMRs, ovarian tissues from three PNA mice and two control mice were assessed by MBD-seq, followed by a comparative analysis of the methylated regions. Detailed information regarding the sequencing data is shown in Supplementary Table S2B. Data quality control is shown in Supplementary Figure S1. There were 17,726 significant DMRs [*p* < 0.05, | log_2_(fold change)| > 1] between the PNA and control mice, 3,600 of which were hypermethylated and 14,126 of which were hypomethylated. Compared to the control mice, these 17,726 DMRs showed significantly different methylation levels in PNA mice, and the hierarchical cluster analysis results also showed that the methylation patterns were different between the PNA mice and control ovaries (Figure 2A). We then assessed the distribution of hypermethylated (red) and hypomethylated (blue) DMRs across the whole genomes. We found that the proportion of hypomethylated DMRs was significantly higher than the proportion of hypermethylated DMRs (Figures 2B,C). Furthermore, we analyzed the distribution of DMRs in CpG islands and genome features. In the CpG islands and the surrounding areas, the proportion of hypomethylated DMRs was significantly higher than the proportion of hypermethylated DMRs. In the promoter regions, the proportion of hypomethylated DMRs was significantly higher than the proportion of hypermethylated DMRs. The ratios between hypermethylated and hypomethylated DMRs were consistent with this overall pattern in most genome features, including the 5′ untranslated regions (UTRs), exons, and coding sequence (CDS) regions, but not the introns and 3′ UTRs (Figure 2D).

Next, we focused on the DMRs in the promoter regions, which may participate in transcriptional regulation. There were 857 DMRs in promoter regions (Supplementary Table S3), comprising 136 hypermethylated genes and 721 hypomethylated genes. Gene Ontology (GO) and Kyoto Encyclopedia of Genes and Genomes (KEGG) enrichment analyses were performed for the 721 DMGs with hypomethylated promoter regions. These DMGs were enriched in several biological processes related to protein signal transduction, such as small GTPase-mediated signal transduction (GO: 0007264), Ras protein signal transduction (GO: 0007265), and positive regulation of protein kinase activity (GO: 0045860) (Figure 2F). They were also enriched in various enzyme activities (some notably involved in metabolic pathways), such as protein serine/threonine kinase activity, phosphoric ester hydrolase activity, and lysosomes (*p* < 0.05). In addition, the top 10 KEGG pathways (in terms of *p*-value) were all related to metabolic function, including the phosphatidylinositol signaling pathway, mitogen-activated protein kinase (MAPK) signaling pathway, and lysosomes (Figure 2E).

### Identification of DEGs and Functional Enrichment Analyses

**FIGURE 3:**
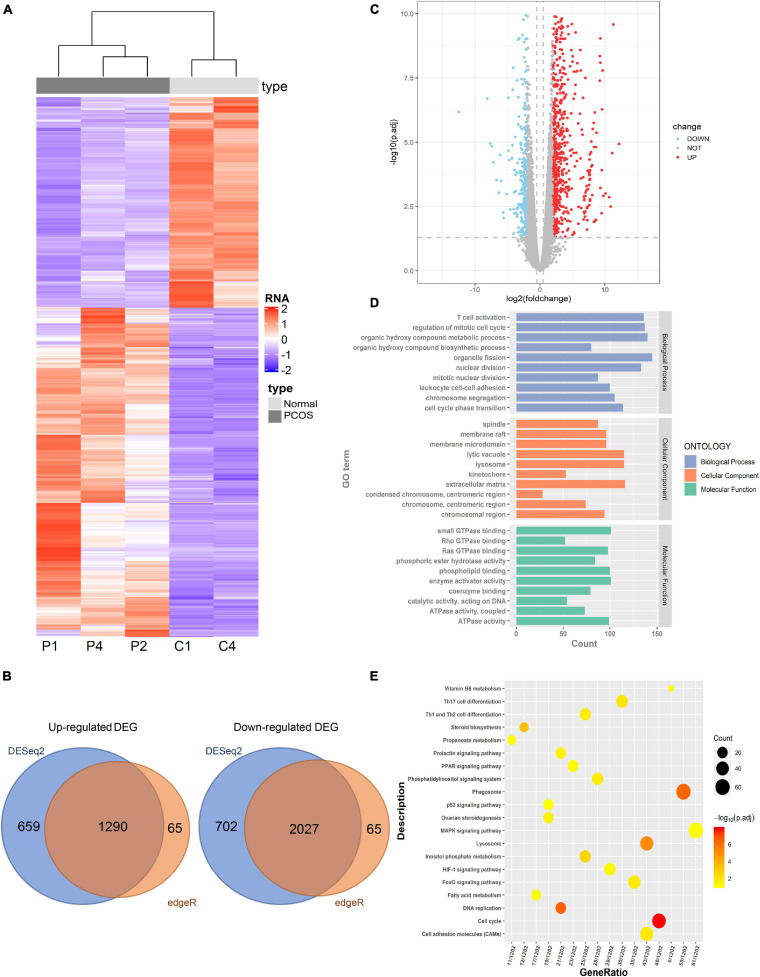
Identification of differently expressed genes (DEGs) between PNA mice’s ovaries and the control group. **(A)** The hierarchical clustering heat map of DEGs in PNA mice (P1, P2, and P4) vs. control group (C1 and C4). **(B)** The volcano map of DEGs in PNA mice vs. control group. **(C)** The Venn map of upregulated DEGs and downregulated DEGs by DESeq2 and edgeR. **(D)** GO analysis of DEGs. **(E)** KEGG analysis of DEGs.

To determine the significant DEGs between the PNA and control mice, the transcriptome profiles of ovaries from three PNA mice and two control mice were assessed by RNA-seq. Detailed information regarding the sequencing data is summarized in Supplementary Table S2C. To accurately identify the DEGs, we focused on the common DEGs identified by two different identification methods (DESeq2 and edgeR). Hierarchical clustering analysis indicated the significantly different expression patterns between the PNA mice and control group (Figure 3A). We identified 3,317 DEGs, comprising 2,027 upregulated genes and 1,290 downregulated genes (Figures 3B,C and Supplementary Table S4). Moreover, we analyzed the regulatory function of the DEGs by using GO and KEGG enrichment analyses. Certain GO biological processes and molecular functions were significantly enriched, such as T cell activation (GO: 0030217), small GTPase binding (GO: 0007264), and Ras protein signal transduction (GO: 0007265) (Figure 3D). The previous study indicated that some specific type T cells as regulators of immune responses might be involved in the pathogenesis of PCOS (Nasri et al., 2018). Moreover, ARF6 small GTPase was observed highly expressed in GC from PCOS patients (Kanamarlapudi et al., 2016). In addition, the KEGG enrichment analysis indicated that the DEGs were involved in the phagosome, steroid biosynthesis, FoxO signaling pathway, the p53 signaling pathway, lysosomes, and the MAPK signaling pathway (Figure 3E). These pathways were involved in the occurrence and development of PCOS in different aspects. Androgen-induced activation of autophagy was considered an important role in the development of PCOS (Li et al., 2019). The P53 signaling pathway and MAPK signaling pathway were participated in the regulation of various metabolic processes including PCOS. Besides, we found a significant downregulation of *Dnmt1*, which may be related to global hypomethylation in PNA mice.

### Identification of Hypomethylated Promoter Regions That Allow DEG Upregulation

**FIGURE 4:**
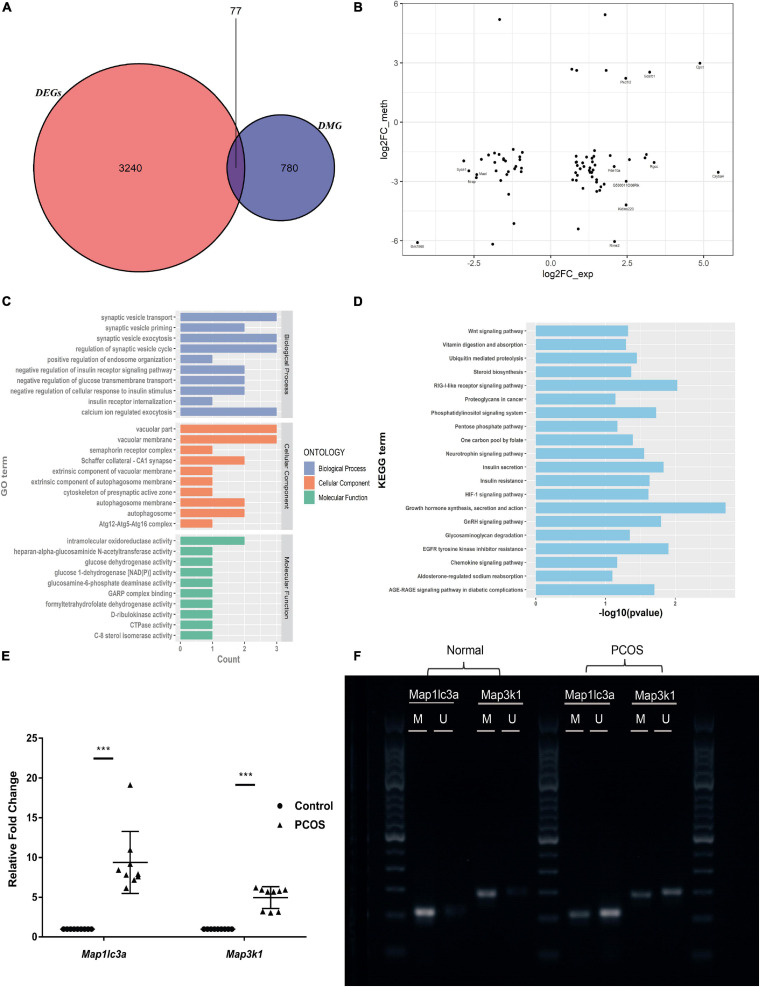
Identification and validation of genes with hypomethylated promoter and which are negatively related with expression. **(A)** The Venn map of DMEGs. **(B)** The dot plots of methylation level and gene expression in the promoter region of DMEGs. **(C)** GO analysis of DMEGs in the hypomethylated promoter with an upregulated expression. **(D)** KEGG analysis of DMEGs in the hypomethylated promoter with an upregulated expression. **(E)** RT-qPCR scatter plots of *Map3k1* and *Map1lc3a* (***p < 0.001). **(F)** MSP agarose gel electrophoresis results.

Aberrant DNA methylation of promoter regions can directly regulate gene expression. Analyzing the 3,317 DEGs and the 857 DMGs with differentially methylated promoter regions, we found that 77 of the DEGs had aberrantly methylated promoter regions (Figures 4A,B). Among those that were hypomethylated, 41 (such as *Map3k1*, *Map1lc3a*, and *Stat3*) were significantly upregulated and one (*Cenpe*) was significantly downregulated (Supplementary Table S5). The functional enrichment analysis of the former genes (i.e., upregulated DEGs with hypomethylated promoter regions) showed that autophagy, growth hormone synthesis, secretion, and action-related pathways were significantly enriched (Figures 4C,D). After functional analysis of DMGs with differentially regulated promoter regions and DEGs, we selected the genes with the same signaling pathway. From among the resultant genes, we identified the upregulated DEGs with hypomethylated promoter regions. Consequently, we obtained seven genes (*Map3k1*, *Map1lc3a*, *Stat3*, *Prkcb*, *Ebp*, *Hgsnat*, and *Synj1*). The functional enrichment analysis of these seven genes showed that the MAPK signaling pathway, autophagosomes, phosphatidylinositol signaling system, and steroid biosynthesis were significantly enriched.

To illustrate the regulatory functions of methylation in PCOS, we focused on *Map3k1* and *Map1lc3a*, which are involved in the MAPK pathway and autophagy, respectively. These genes were found to have hypomethylated promoter regions which accompanied upregulated expression. Subsequently, using RT-qPCR and MSP, we validated their changes in expression and methylation levels, respectively (Figures 4E,F). Compared to the control mice, in the PNA mice, they were significantly upregulated and their promoter regions showed significant hypomethylation. Thus, *Map3k1* expression and its promoter methylation level were negatively associated, and this was also the case for *Map1lc3a*.

### Autophagy Profiling and Assessment of Gene Expression Related to the MAPK Pathway and Autophagy in PNA Mice’s Ovaries

**FIGURE 5:**
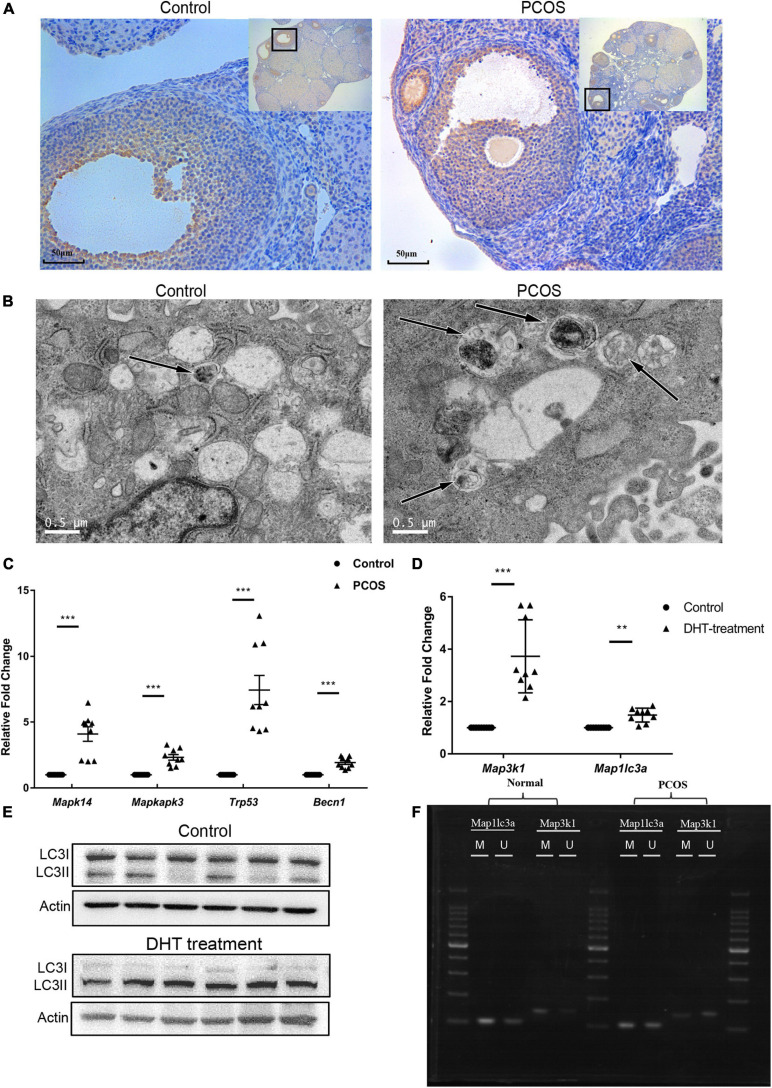
Validation of the expression of MAPK signaling pathway and autophagy-related genes. **(A)** Immunohistochemistry of LC3II in PNA ovarian tissue and control groups. **(B)** Autophagosome detection under a transmission electron microscope. **(C)** RT-qPCR scatter plots of MAPK signaling pathway and autophagy-related genes *Mapk14*, *Mapkapk3*, *Trp53*, and *Becn1* (***p < 0.001). **(D)** RT-qPCR scatter plots of *Map3k1* and *Map1lc3a* in DHT-treated granulosa cells and control group (**p < 0.01, and ***p < 0.001). **(E)** Protein expression of LC3II and LC3I in the DHT-treated granulosa cells and control group. **(F)** MSP agarose gel electrophoresis results of *Map3k1* and *Map1lc3a* in DHT-treated granulosa cells and control group.

*Map1lc3a* encodes LC3II, an autophagy marker. To investigate changes in autophagy in PNA mice’s ovaries, we performed immunohistochemical staining using the anti-LC3II antibody in the ovarian tissues of three PNA mice and three control mice (representative results are shown in Figure 5A). The LC3II protein expression in the follicles was significantly higher in the PNA mice than the control mice. Subsequently, autophagosomes in the granulosa cells of PNA and control mice were detected by TEM, and the results showed an increased number of autophagosomes in the PNA mice, which indicated a significant increase in autophagy in the ovaries of the PNA mice (Figure 5B). To investigate the regulatory relationship between the MAPK pathway and autophagy in PNA mice’s ovaries, we performed RT-qPCR on three MAPK/p38 pathway-related genes, *Mapk14* (P38), *Mapkapk3*, and *Trp53* (P53), and the autophagy-related gene *Becn1*, in the ovarian tissues of three PNA mice and three control mice. The results showed that the expression levels of all four genes were significantly upregulated in the PNA mice (Figure 5C).

### The Effect of DHT Treatment on Expression and Methylation of *Map3k1* and *Map1lc3a* and Autophagy Level in Granulosa Cells

To investigate whether DHT treatment is directly related to the expression and methylation level change, the RT-qPCR and MSP of *Map3k1* and *Map1lc3a* were performed on DHT treatment granulosa cells (*n* = 6) and control group (*n* = 6). To characterize changes in autophagy, LC3II was detected on the DHT-treated and untreated cells also. The GCs were first treated with DHT with different concentrations (0, 1, or 2 μmol/L) for 24 and 48 h for our experiments. There were no significant differences between DHT treated and untreated cells for 24 h. However, the expression level of *Map3k1* and *Map1lc3a* was significantly upregulated accompanied by the methylation level downregulated of *Map3k1* in the 1-μmol/L DHT-treated group after 48 h. The methylation level change of *Map1lc3a* was not significant, which means that a change in the methylation of *Map1lc3a* may be caused by comprehensive factors and a more complex regulation in PNA mice (Figures 5D,F). Meanwhile, the expression of LC3II was significantly increased, which means the autophagy level increased in the DHT-treated group (Figure 5E). The results showed that DHT is the direct cause of *Map3k1* expression and the methylation level changed, which means DHT may directly cause the activation of the MAPK signaling pathway by regulating the methylation and expression levels of *Map3k1* and then affected the autophagy level in granulosa cells.

## Discussion

Epigenetic modifications, such as DNA methylation, regulate gene expression in a variety of important physiological and pathological processes, including cell proliferation, apoptosis, inflammation, and carcinogenesis (Koch et al., 2018). DNA methylation is reported to be a major factor in the development of PCOS (Edgar Ricardo et al., 2019). Recent research has shown that CpG hypomethylation regarding *GHRHR*, *AKR1C3*, *RETN*, and *MAMLD1* and hypermethylation regarding *TNF*, which can indirectly contribute to androgen excess, were consistent with increased and decreased levels of the corresponding gene transcripts, respectively (Sagvekar et al., 2019). In addition, research has shown that the children of women with PCOS show a sex-specific pattern of DNA methylation in the promoter regions of genes associated with the reproductive and metabolic features of PCOS at early infancy (Echiburú et al., 2020). However, the specific effects of the DNA methylation on the associated gene expression and downstream metabolism remain unknown.

Using MBD-seq, we identified 857 DMGs with differentially methylated promoter regions between PNA and control mice. The DMGs with hypermethylated promoter regions were enriched in KEGG pathways such as the phosphatidylinositol signaling pathway, lysosomes, and the MAPK signaling pathway (Figure 2E). The KEGG enrichment analysis of the 3,317 DEGs that we identified by RNA-seq showed that the DEGs were enriched in immune-related and metabolic pathways, such as the FoxO signaling pathway, p53 signaling pathway, steroid biosynthesis, lysosomes, and the MAPK signaling pathway (Figure 3E). Hence, it was reasonable to hypothesize that the abnormal DNA methylation affected gene expression related to the MAPK signaling pathway and autophagy.

We found that the PNA mice had downregulation of *Dnmt1*, accompanied by global hypomethylation, compared to the control mice. By combining two types of sequencing data (RNA-seq and MBD-seq) and validation (using RT-qPCR and MSP), we found that the expression levels of *Map3k1* (which encodes MEKK1, a key enzyme in the MAPK pathway) and *Map1lc3a* (which encodes LC3I/LC3II, the latter of which is involved in autophagy) were both negatively correlated with the methylation level of their respective promoter.

*Map3k1* encodes the serine–threonine kinase MEKK1 (Pham et al., 2013), which belongs to the mitogen-activated protein kinase kinase kinase 1 (MAP3K1) family (Hagemann and Blank, 2001). In this family, MAP3K1 is the only member that contains specific domains and features (caspase cleavage site and E3 ligase activity) (Xia et al., 2007). *Map3k1* is activated by a variety of cell stresses and other stimuli such as growth factors, cytokines, microtubule disruption, and cold temperature, and it activates various downstream effector molecules in the MAPK pathway (including JNK, ERK1/2, and P38), performing a regulatory role. Thus, *Map3k1* appears to play a critical role in balancing cell fate decisions (Lange-Carter et al., 1993; Minden et al., 1994). Research has shown that in PCOS, the MAPK signaling pathway can be activated due to various reasons, such as oxidative stress and chronic inflammation, thereby participating in the regulation of PCOS-related metabolism and disease development (Zhao et al., 2015; Zuo et al., 2016). In our study, the increased expression caused by hypomethylation of the *Map3k1* promoter increased the expression levels of *Mapk14* (P38), *Mapkapk3*, and *Trp53* (P53), which indicated the activity of the MAPK/P38 signaling pathway. In cell experiments, a significant upregulation of *Map3k1* accompanied by methylation level downregulated was observed in DHT-treated granulosa cells. The results illustrated that DHT is a direct cause of the changes in *Map3k1* expression and methylation level, which is considered to be the important cause of activation of MAPK pathway and autophagy.

*Map1lc3a* encodes microtubule-associated protein 1 light chain 3 alpha (LC3), which is divided into the cytosolic form of LC3 (LC3I) and the LC3-phosphatidylethanolamine conjugate (LC3II). LC3II reflects autophagic activity (Runwal et al., 2019). In addition, the autophagy-related gene *Becn1* is widely used for monitoring the autophagy process. *Becn1* is necessary for the initiation of autophagy, playing an important role in autophagosome formation (Hill et al., 2019). Usually, autophagy involves a high expression of the LC3II protein and autophagy-related proteins and a decrease in the autophagy substrate P62. Autophagosome detection under TEM is the gold standard for verifying changes in autophagy levels. Autophagy is a major catabolic pathway that affects cell survival, differentiation, tumorigenesis, and neurodegeneration (Sun et al., 2017). Autophagy was significantly increased in the PNA mice compared to the control mice, especially in granulosa cells, which is consistent with the manifestations of PCOS in humans in previous research (Li et al., 2018). Besides, a high concentration of DHT can directly increase autophagy in granulosa cells according to our experiments. Autophagy may be an important molecular event in the pathogenesis of PCOS.

MEKK1 is an important component upstream of the MAPK signaling pathway, and it was significantly upregulated in the PNA mice due to hypomethylation of its promoter region. The downstream proteins P38 and P53 can regulate autophagy, leading to upregulation of LC3II and autophagy-related proteins. In many studies of cancer and PCOS, the MAPK signaling pathway has been shown to play an important role in regulating autophagy levels and participating in disease processes. Research has also shown that there is an obvious autophagy disorder in PCOS patients’ granulosa cells, which may relate to the follicular atresia and metabolic disorders observed in PCOS patients. In PNA mice’s ovaries and DHT-treated granulosa cells, insufficient methylation of the *Map3k1* promoter region leads to MAPK pathway activation, which in turn triggers a series of downstream reactions, leading to a significant increase in autophagy.

Despite that the DHT-induced PNA mice effectively simulated the physiological state of hyperandrogenemia and are suitable for PCOS tissue morphology and metabolism research, there are some limitations of the model. First, the level of luteinizing hormone (LH) in the serum of the DHT-induced model was not increased but decreased, which was not consistent with clinical features of PCOS patients. Furthermore, the DHT-induced PNA model lacked a neuroendocrine phenotype, which manifested in non-significantly different anti-Mullerian hormone (AMH) expression levels in the DHT-induced PNA model and control group (Benrick et al., 2017). MBD-seq is a common approach for methylome-wide association studies (MWAS) that provides near-complete coverage of the methylome at similar costs as the array-based technologies. Compared with the whole-genome bisulfite sequencing (WGB-seq), which based on bisulfite conversion and had the methylation status of each CpG site, MBD-seq lacked coverage for unmethylated/hypomethylated CpGs and single-site resolution information (Aberg et al., 2020), which were also the disadvantage of MSP. Besides, MBD-seq is specific for CpG methylation (mCG), which means it will not identify methylation outside the sequence context of CG (mCH) nor hydroxymethylation (hmC). Thus, to investigate these methylation types in the association study, MBD-seq can be complemented with enrichment approaches for mCH [MBD-DIP (Chan et al., 2017)] and hmC [hMe-Seal (Song et al., 2011)]. Due to the limited number of samples, more extensive experimental exploration is required to verify the findings of the comprehensive analysis of DNA methylation and autophagy-related pathways in this study. In particular, to better understand the role of epigenetic regulation in the expression of PCOS-related genes, it is necessary to further study how various epigenetic regulatory factors influence each other. This should be combined with exploring the pathogenesis of PCOS, including the regulatory function of granulosa cells, in clinical samples. This could help to develop new treatment strategies and to identify diagnostic and prognostic markers.

## Conclusion

Factors underlying PCOS can downregulate *Dnmt1* expression, which may lead to abnormal methylation of the MAPK pathway-related gene *Map3k1* and the autophagy-related gene *Map1lc3a*. We observed that in the PNA mice’s ovaries, autophagy was significantly increased, especially in granulosa cells. *In vitro*, excessive androgen such as DHT would change the DNA methylation and transcription level of *Map3k1* and lead to autophagy increased in granulosa cells directly. The overall autophagy disorder may be driven by DNA methylation and transcriptional changes regarding the MAPK pathway-related gene *Map3k1*. Our results indicate that the epigenetic imbalance may cause the activation of the MAPK signaling pathway, triggering a series of downstream responses, which are of great significance for follicular hypoplasia, hyperautophagy, and the metabolic disorders of PCOS. Our study provides a novel genetic basis and new insights regarding the pathogenesis of PCOS.

## Data Availability Statement

The datasets presented in this study can be found in online repositories. The names of the repository/repositories and accession number(s) can be found in the article/Supplementary Material.

## Ethics Statement

All of the experimental procedures were performed in accordance with the guidelines of the Experimental Animals Management Committee (Jiangsu Province, China) and were approved by Nanjing Drum Tower Hospital Experimental Animals Welfare Ethical Committee (20150302).

## Author Contributions

YK and TL conceived and designed the study. YQ and HZ performed the experiments. HZ and CD analyzed the data. YQ and ZM wrote the manuscript and figures. YK, TL, and CD revised the manuscript. All authors read and approved the final manuscript.

## Conflict of Interest

The authors declare that the research was conducted in the absence of any commercial or financial relationships that could be construed as a potential conflict of interest.

## Footnotes

1 https://www.ncbi.nlm.nih.gov/geo/

## Supplementary Material

Supplementary Figure 1 Quality control of MBD-seq data.

Supplementary Figure 2 Electron micrographs in lower magnifications of granulosa cells.

Supplementary Table 1 PNA mouse model identification data.

Supplementary Table 2 Primer sequence and sequencing data information. **(A)** Primer sequences. **(B,C)** Sequencing data information of MBD-seq and RNA-seq in PNA mice vs. control group.

Supplementary Table 3 Differently methylated promoters in PNA mice vs. control group.

Supplementary Table 4 Differently expressed genes in PNA mice vs. control group.

Supplementary Table 5 Promoter-methylated genes with negative related expression in PNA mice vs. control group.
