# Supplementary figures and images for: Integrated Transcriptomic and Epigenetic Study of PCOS: Impact of Map3k1 and Map1lc3a Promoter Methylation on Autophagy
Source: Front Genet. 2021 Mar 8;12:620241. doi: 10.3389/fgene.2021.620241 (PMC7982605; doi:10.3389/fgene.2021.620241)

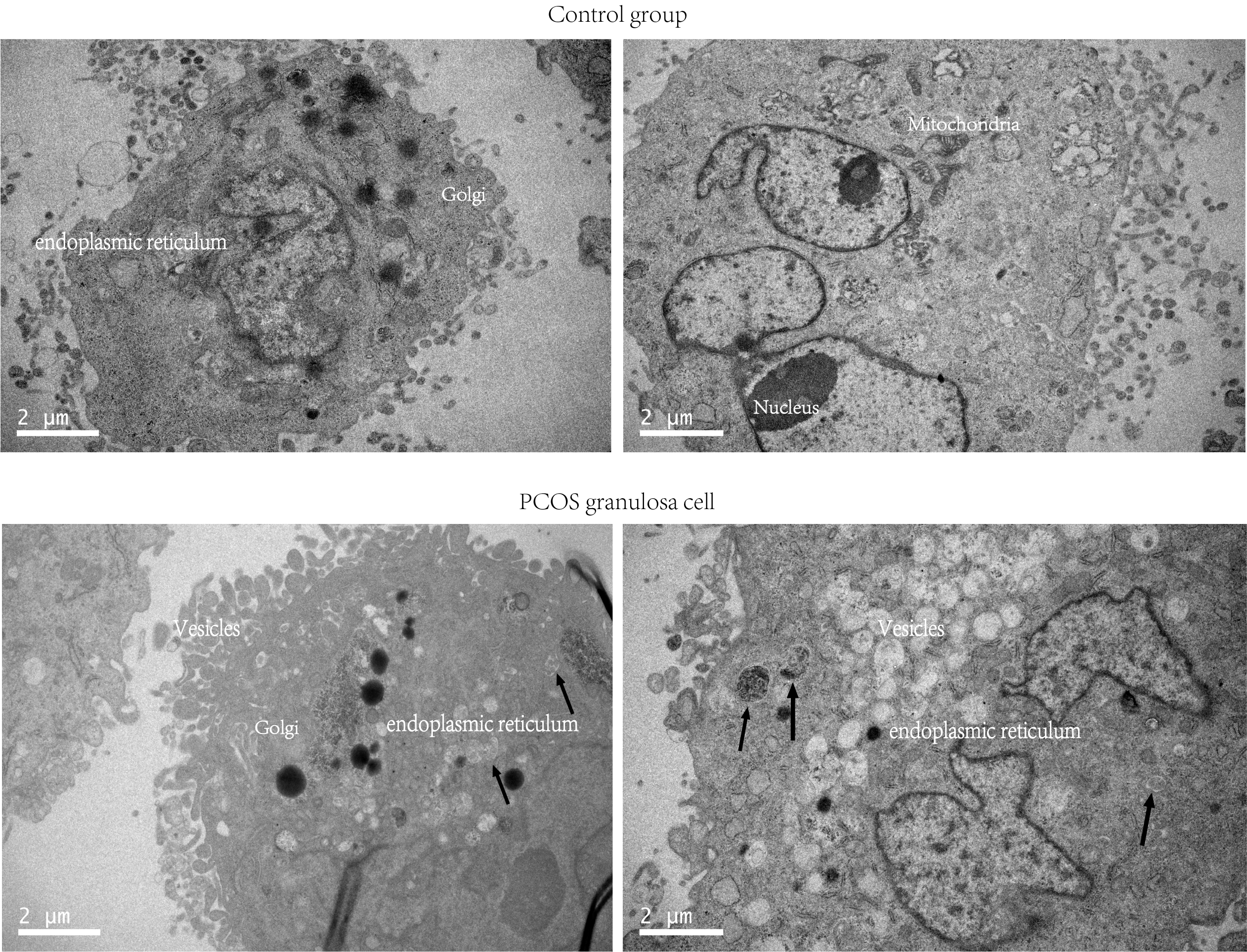

Supplement: Supplementary Figure 1 — Quality control of MBD-seq data. [file Image_2.tif]

## Quality Control 1: data coverage

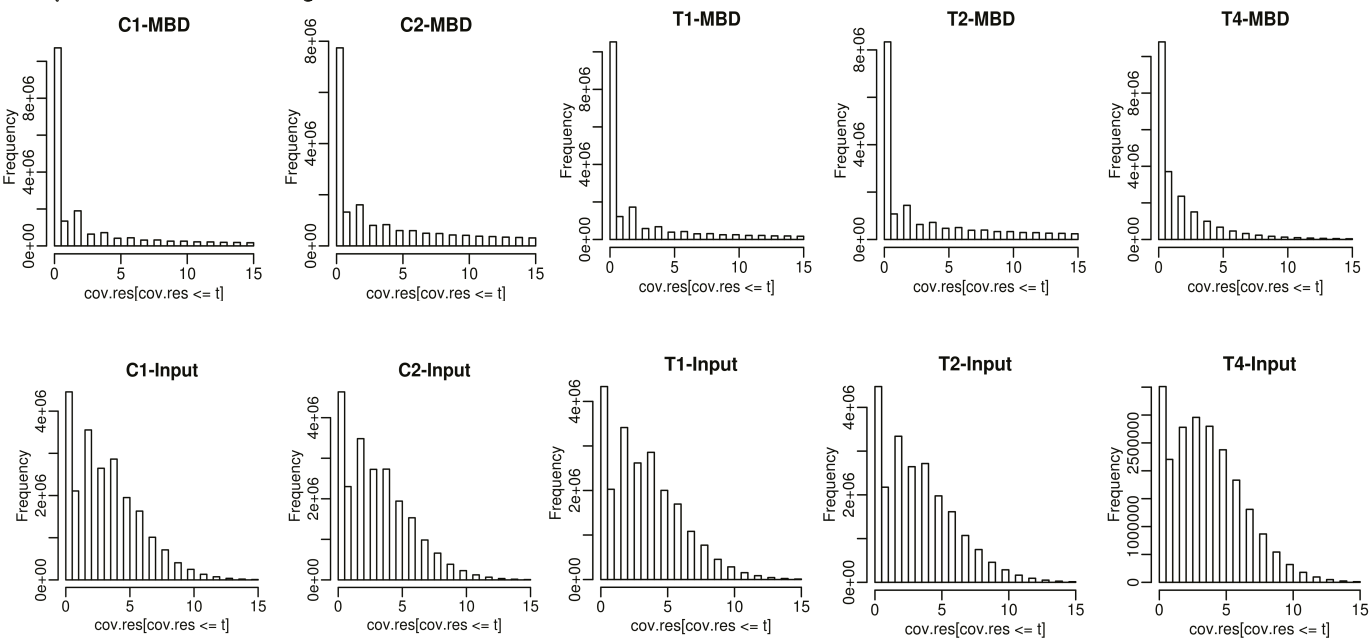

## Quality Control 2: data Saturation

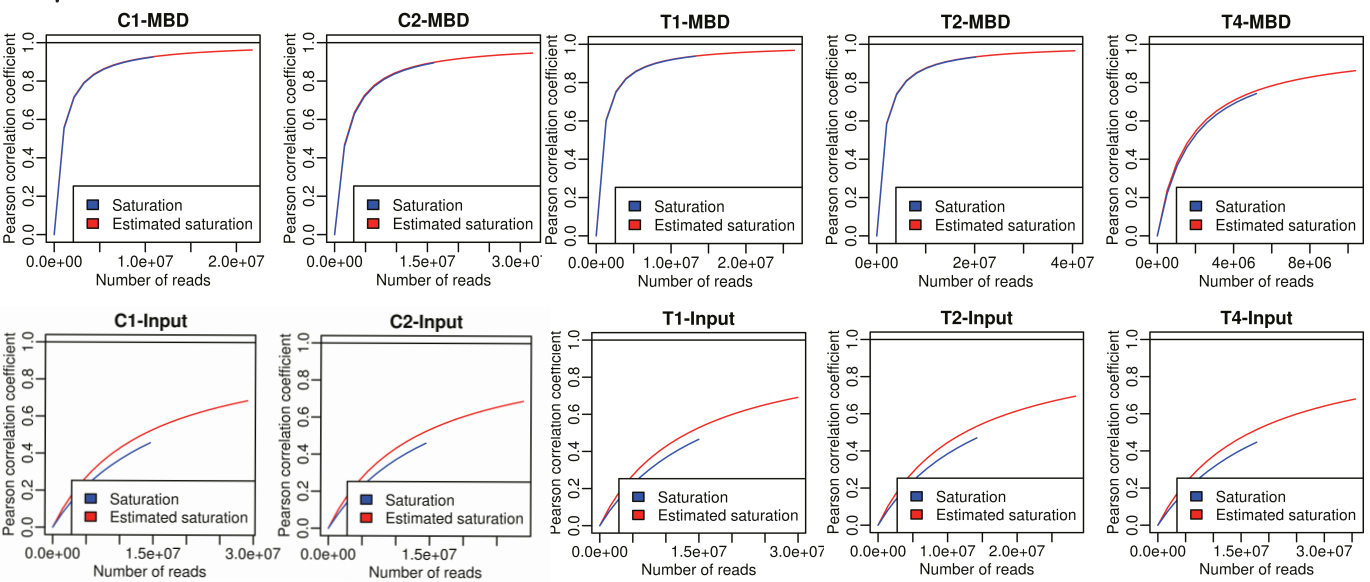

Supplement: Supplementary Figure 2 — Electron micrographs in lower magnifications of granulosa cells. [file Image_1.pdf]
